# Astrocyte-Synapse Structural Plasticity

**DOI:** 10.1155/2014/232105

**Published:** 2014-01-08

**Authors:** Yann Bernardinelli, Dominique Muller, Irina Nikonenko

**Affiliations:** Department of Neuroscience, School of Medicine, University of Geneva, Geneva, Switzerland

## Abstract

The function and efficacy of synaptic transmission are determined not only by the composition and activity of pre- and postsynaptic components but also by the environment in which a synapse is embedded. Glial cells constitute an important part of this environment and participate in several aspects of synaptic functions. Among the glial cell family, the roles played by astrocytes at the synaptic level are particularly important, ranging from the trophic support to the fine-tuning of transmission. Astrocytic structures are frequently observed in close association with glutamatergic synapses, providing a morphological entity for bidirectional interactions with synapses. Experimental evidence indicates that astrocytes sense neuronal activity by elevating their intracellular calcium in response to neurotransmitters and may communicate with neurons. The precise role of astrocytes in regulating synaptic properties, function, and plasticity remains however a subject of intense debate and many aspects of their interactions with neurons remain to be investigated. A particularly intriguing aspect is their ability to rapidly restructure their processes and modify their coverage of the synaptic elements. The present review summarizes some of these findings with a particular focus on the mechanisms driving this form of structural plasticity and its possible impact on synaptic structure and function.

## 1. Introduction

Since the earliest studies on glial cells in the 19th century, Ramón y Cajal, Camillo Golgi, and their contemporary colleagues have described astrocytes as very particular cells in intimate contact with neurons and capillaries. Based on these observations, they made different hypotheses on their physiological function, ranging from passive space filling in the neuropil to active energy supply for neurons [1]. Almost 150 years later, the neurophysiological role of astrocytes is still a subject of intense debate, although increasing data suggest that they are active players in mechanisms of synaptic transmission and plasticity [2]. Numerous data demonstrate that thin astrocytic processes infiltrate brain tissue [3]. The most commonly used name for these thin processes is “peripheral astrocytic processes,” as it is often difficult to distinguish, with light microscopy, their exact position with regard to different neuropil elements. However, in this review we will mostly focus on the data, obtained with various techniques, concerning fine astrocytic processes that are in close association with synaptic contacts, and thus the term “perisynaptic astrocytic process” (PAP [4]) will be applied throughout the review.

Close structural relationship between synaptic structures and PAPs makes astrocytes an important partner of neurons in the organization and functioning of synaptic connections. Astrocytes take up glutamate from the synaptic cleft [5], control the amount of glutamate spillover that activates extrasynaptic receptors or enables intersynaptic crosstalk [6], control the ion and water homeostasis through selective transmembrane movements of inorganic and organic molecules and the equilibration of osmotic gradients [7], and provide energy substrates to neurons in the form of lactate [8]. In addition, recent studies have revealed astrocytic processes as structural entities able to modulate neuronal function at the synaptic level through the calcium-dependent release of gliotransmitters [9, 10]. Indeed, astrocytes are able to sense neuronal activity by elevating their intracellular calcium concentration through Gq-coupled protein mechanisms [11]. Even if this calcium excitability phenomenon is well accepted, its exact role is still controversial [12]. Finally, PAPs have been described by several studies as plastic structures able to change their morphology within minutes, thus modifying their coverage of pre- and postsynaptic elements [13]. We review here these observations and discuss the physiological role that structural changes in PAPs could have on both synaptic and astrocytic function.

## 2. Astrocytic Processes Embrace Synapses

**Figure 1:**
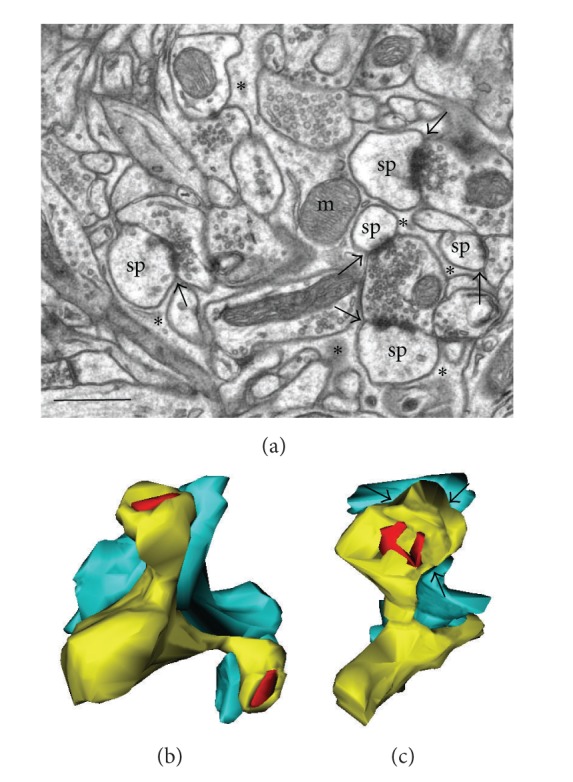
Electron microscopy images and 3D reconstructions illustrating glial coverage of excitatory spine synapses in neocortex. (a) EM micrograph of the neuropil in the adult mouse cortex with dendritic spine synapses (sp) surrounded by astrocytic processes marked with asterisks. Note mitochondria (m) in the branching point of astrocytic processes and perisynaptic astrocytic processes (PAPs) contacting axon-spine interface (ASI, arrows). (b) 3D reconstruction of the PAP near spine synapses with simple PSDs in adult rat hippocampus. Dendritic segment with two spines is in yellow, PSDs are in red, and PAP is in blue. (c) The same but for the big spine with complex PSD. Arrows point at ASI covered by the PAP. Note that in both reconstructions a large fraction of ASI is devoid of PAPs (original data). Scale bar: 1 *μ*m.

A main structural characteristic of astrocytes is the star-shaped arborization of their stem processes. However, at the ultrastructural level, protoplasmic astrocytes have much more complex shapes consisting of thousands of very fine elaborated protrusions that make them look more like sponges than stars [14–16]. Detailed morphological descriptions and quantitative analyses provided by electron microscopy (EM), especially those based on three-dimensional (3D) reconstructions from serial ultrathin sections, have allowed the identification of astroglia and their interactions with neurons and synapses. Big stem processes of astrocytes containing typical bundles of intermediate filaments (8-9 nm in diameter) that have glial fibrillary acidic protein (GFAP) as their main constituent represent around 15% of the total volume of an astrocyte [17]. These processes ramify progressively to finally generate a dense matrix of thin elaborate terminal processes, which infiltrate brain tissue and closely associate with neuropil elements and particularly with synapses. These fine astrocytic processes account for 70–80% of the astrocytic plasma membrane [18] and often surround spine synapses, sometimes completely encapsulating them (Figure 1). Astrocytic processes are found in close proximity of synapses at all synapses investigated so far with EM in different brain regions. These small astrocytic processes are devoid of GFAP filaments, have a cytoplasm that is light in appearance, are highly irregular in outline, forming flat or lamellar sheets or conforming to the shapes of surrounding neuropil elements (Figure 1), and have large surface/volume ratios, as shown with 3D EM reconstructions [19, 20] (reviewed in [13]). In these very fine processes, mitochondria, microtubules, and endoplasmic reticulum are usually absent, but ribosomes and glycogen granules are common [21]. They also contain actin filaments [22], together with actin-binding proteins [4, 23, 24]. The contacts between astrocytic processes and dendritic spines can be quite tight, sometimes with puncta adhaerens between the nonsynaptic surface of dendritic spines and astrocytic processes [25].

PAPs are found in all brain regions, but the proportion of synapses having them and the level of synaptic coverage vary significantly. For example, PAPs embrace most of the synapses in cerebellum but only 29–56% of the synapses in the neocortex, according to different EM estimations [18, 26]. In layer IV of adult mouse somatosensory cortex, 3D EM demonstrated high heterogeneity of synaptic coverage, with around 10% of spine synapses having no contact with PAPs, while the rest of the spines have varying portion of their surface enwrapped, with a majority of them (around 68%) surrounded by astrocytic process at the axon-spine interface (ASI; Figure 1). In CA1 stratum radiatum of adult rat hippocampus, PAPs are also distributed nonuniformly. They are present near approximately 62% of synapses, with a preference towards large synapses, especially those with perforated (complex) PSDs that are considered as morphological correlates of strengthened synapses (up to 90%; [27]). Some CA1 synapses are completely devoid of PAPs, while others could be covered only around the dendritic spine (7%), or around the presynaptic bouton (8%), or, most often, around the ASI. In CA1 area of rat organotypic hippocampal slice cultures, the majority (85%) of spine synapses are also contacted by glial processes that do not cover synaptic surface completely. 97% of big spine synapses with a complex PSD are partially enveloped by glia but only 78% of simple synapses [28]. In rat hippocampus, less than half (43%) of the synaptic perimeter is surrounded by PAPs, and this parameter is highly variable from synapse to synapse ([29]; see Figures 1(b) and 1(c)). In general, 99.5% of CA1 synapses have some portions of ASI free from glial processes, and only a fraction of the ASI perimeter (on average, 38%) is enwrapped by astroglia. In contrast, in CA3 hippocampal region, large mossy fiber synapses are surrounded by astrocytic processes that physically isolate these synaptic complexes from the neighboring synapses, although PAPs never reach individual active zones [30].

In cerebellum, a mossy fiber (MF) excitatory terminal has hundreds of release sites that establish synaptic contacts with different granule cell dendrites positioned close together forming a multisynaptic arrangement or glomerulus. Only a small fraction (around 15%) of these excitatory terminals is contacted by glial processes, as shown by 3D EM [31]. In contrast, excitatory inputs to Purkinje cell (PC) spines are much better isolated by the Bergman glia (BG) processes [32]. Synapses formed by climbing fibers (CF) have the highest level of astrocytic coverage, with an average degree of enwrapping around 87%. In more than half of these synapses, at least 90% of their surface is covered by PAPs. Parallel fiber (PF) synapses have a bit less of perisynaptic glial coverage (around 65% of the synapse perimeter [32]). Serial 3D EM of labeled lateral BG processes that represent up to 90% of the BG total membrane surface area demonstrated their very complex and irregular morphology, with high surface to volume ratio [19]. Another peculiar morphological organization revealed by serial 3D EM of labeled lateral BG processes is the so-called BG microdomain, a repetitive unit on the stem process consisting of a thin stalk and a complex cabbage-like head packed with thin leaflets that wrap around individual synapses or groups of synapses and constitutes an autonomous functional unit.

In the Nucleus Tractus Solitarii (NTS), a sensory nucleus in the caudal medulla that receives primary afferent visceroceptive inputs, but also afferent fibers from various brain regions, volume fraction of astrocytic processes and the density of astrocyte membranes are rather high, equal to 15% and 2.8 *μ*m^2^/*μ*m^3^, respectively. This nucleus contains simple one-to-one, mostly axodendritic, synapses but is also enriched in divergent (one axonal terminal innervating several postsynaptic dendrites or spines) and convergent (several axon terminals making clustered synaptic contacts on the same dendritic segment) multisynaptic arrangements or glomeruli. Like in other brain regions, astrocytic coverage of synapses is generally not complete and highly variable, ranging from no coverage to almost complete enwrapping, and constitutes, on average, 47% of synapse perimeter. At simple glutamatergic synapses, astrocytic processes cover 58% of their perimeter, while, in multisynaptic arrangements, glial coverage is restricted to the nonshared, outer part of synaptic perimeters and amounts around 50% of this outer part [33]. For more examples of PAP distribution and synaptic ensheathment see also [3, 13].

Although electron microscopy provides the most precise estimations of PAP morphology, distribution, localization, and degree of synaptic coverage, this technique is limited to static observations as it requires fixation and embedding of tissue samples. Current light microscopy approaches use PAP labeling with different fluorescent probes, static or time-lapse observations with confocal microscopy followed by off-line image treatment (e.g., deconvolution helping to overcome the limits of confocal resolution, often lower than the size of the smallest PAPs), and 3D/4D reconstructions [34, 35]. These data will be discussed in the following sections. Development of superresolution light microscopy imaging techniques [36] will further improve the precision of PAP observations.

## 3. PAP Distribution Is Related to the Patterns of Neuronal Connectivity in Different Brain Regions

The distribution of astrocytic processes shows high variability between different brain regions and even subregions. One of the first indications was provided by a light microscopy study that assessed the orientation of hippocampal astrocytic processes [37]. They found that during development, hippocampal astrocytes become polarized. Their orientation in CA1 is almost perpendicular to the stratum pyramidale, while in stratum lacunosum-moleculare they transiently orient parallel to the fissure. This particular orientation pattern seems to correlate with neuronal projections in the hippocampus [37].

Confocal and electron microscopy studies in hippocampus [17, 38] and neocortex [15] have shown that astrocytes cover their own exclusive spatial domains where they interact with neuronal elements, with only limited overlap in narrow peripheral zones. This typical domain organization helps the demarcation of functional compartments and adequate neurovascular regulation. However, in some cortical regions this organization is compromised. In posterior piriform cortex (PPC) of adult rats, surface-associated astrocytes (SAA) are directly opposed to the cortical surface, send large-caliber processes into layer I, and give rise to an extensive network of superficial processes that form a continuous sheet at the surface of PPC and lack the domain organization typical of neocortical astrocytes [39]. In PPC, connectivity and patterns of activation by individual odorants are widely distributed, overlap extensively, and are modulated by context and behavior, and thus SAA also form extensively overlapping processes [39].

In hippocampus, 99.5% of CA1 synapses have some portions of ASI free from glial processes, thus favoring glutamate spillover and synaptic crosstalk. Interestingly, the fraction of synaptic perimeter covered with PAPs is inversely proportional to the synapse size, so that large synapses, often with complex PSDs, have more of ASI open and are more susceptible to the transmitter spillover ([27, 29]; see Figures 1(b) and 1(c)). Importantly, only 33% of the neighboring synapse pairs in the neuropil have an astrocytic process intruding along the shortest path between them, thus further favoring spillover and crosstalk [29]. In CA1 synapses, a significant asymmetry characterizes the distribution of perisynaptic glia with PAPs being threefold more present on the postsynaptic side than on the presynaptic side [40]. 3D modeling using these EM data predicted that extrasynaptic actions of glutamate near these synapses would favor presynaptic feedback and preserve specificity transfer of information to the postsynaptic site.

In cerebellum, the morphology of mossy fiber divergent glomeruli, mostly without glial fingers between the granule cell dendrites contacting an MF excitatory terminal, favors glutamate spillover. This organization enhances the efficacy of fast synaptic transmission and contributes to the time course of MF synaptic currents and to the desensitization of postsynaptic AMPA receptors (AMPAR) during short-term depression [31]. Bergman glia constitutes a large volume fraction in cerebellum (33%), more than three times that of hippocampus and cortex [29, 40, 41]. Unlike neocortex and hippocampus, functional microdomains from different BG cells intermingle within a given space of the neuropil [19, 42]. Intermingling of microdomains from different BG cells covering PF and CF synapses may provide an independent microenvironment designed to compartmentalize calcium signals, contribute to independent activation of individual dendritic spines or ensembles of spines on PC, and ensure selectivity in synapse modulation and efficient clearance through glutamate transporters expressed in the fine BG processes [19, 43].

In the NTS synapses, incomplete glial ensheathment of all synaptic types would provide enough routes for glutamate spillover, although the crosstalk between distant synapses is probably reduced, due to the low synaptic density and high volume fraction of astrocytic processes. At the same time, individual synapses in multisynaptic glomeruli in NTS can strongly influence each other thanks to the direct apposition of neighboring synaptic elements mostly devoid of intervening glial processes that generally cover nonshared, outer part of synaptic perimeters. Consequently, these glomeruli may be viewed as individual computing units providing processing of visceral information already at this level, especially given that sensory afferent terminals in the NTS are frequently involved in divergent arrangements. On the other hand, convergent multisynaptic arrangements may also add to information processing by occlusion phenomenon between sensory inputs converging onto the same NTS neuron [33].

These examples demonstrate that the astrocytic spatial organization varies in different brain regions and may be related to the corresponding patterns of neuronal connectivity helping to establish specific functional and structural architectures. This suggests that astrocytes show sensitivity to neuronal activity and could adapt their morphology to the activity in their environment.

## 4. PAPs Exhibit Significant Structural Plasticity

The heterogeneity of PAPs around different synapses described above suggests the ability of astrocytes to control the level of synaptic coverage and, consequently, implies that PAPs might be as plastic as their neuronal counterparts.

Indeed, the first examples of PAP structural plasticity were described a long time ago, in EM studies of oxytocin (OXT) secreting magnocellular neurons in supraoptic (SON) and paraventricular (PVN) nuclei of hypothalamus [44]. Under basal conditions, somata and dendrites of magnocellular secretory neurons are mostly separated by neuropil elements and especially by fine processes of both stellate and radial-like astrocytes [45–47]. Under conditions of stimulation, including parturition, lactation, osmotic stimulation, and stress, astrocytes respond by rapid, within few hours, and reversible structural remodeling leading to a reduced coverage [46, 48]. Even more striking rhythmic ultrastructural rearrangements take place in suprachiasmatic nucleus (SCN) of the rat hypothalamus where glial and neuronal structural plasticity follows 24 hours light/dark (L/D) cycles [49]. These observations revealed an important potential for structural plasticity in astrocytes.

**Figure 2:**
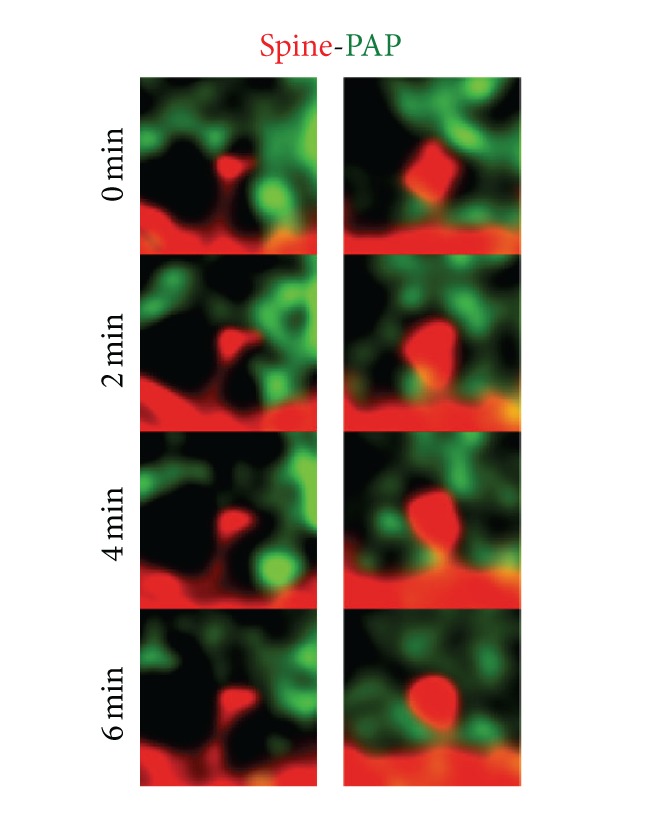
Perisynaptic astrocytic processes (PAPs) are motile. Z-stack projection of confocal images showing hippocampal CA1 dendritic spines (mcherryf, red) and PAPs (eGFPf, green) recorded in the stratum radiatum of organotypic hippocampal slice cultures. Neurons and astrocytes were virally infected with membrane targeted fluorescent proteins by injection. The labeled structures were monitored every 2 min to reveal PAP movements around spines. Square size: 3 × 3 *μ*m (original data).

A technique of choice to observe real time movements of astrocytes is time-lapse light microscopy. In cultured hippocampal or cortical astrocytes, highly motile astrocytic filopodia-like processes were detected, moving or growing in the time course of minutes or even seconds [4, 24, 50]. Whether these filopodia-like structures are comparable to *in situ* PAPs is uncertain, although they contain the actin binding protein ezrin that is specifically localized to PAPs [4]. Thus, the mechanisms driving filopodia movements in cultured astrocytes could be common with those of PAPs *in vivo*. Using a transgenic mice line expressing green fluorescent protein (GFP) under a GFAP promoter, Hirrlinger et al. were able to image astrocytic processes adjacent to synapses in acute brain slices and demonstrate that these processes were motile [51]. However, because of the use of a nonmembrane targeted GFP that limited the possibility to detect very fine astrocytic processes in this and other studies [52, 53], it was difficult to identify whether these processes were indeed PAPs. *In situ* studies using membrane targeted fluorescent protein transfected biolistically in organotypic cultures allowed to visualize very fine astrocytic structures in a time-lapse manner and confirmed that those structures were dynamic [14]. Unfortunately, in this study astrocytic membranes were not coimaged with synaptic elements, again limiting a conclusion as to whether these processes were indeed PAPs. Haber and coworkers were the first to demonstrate convincing movements of PAPs *in situ* by performing dual labeling of dendritic spines and astrocytic processes through viral gene delivery of membrane targeted fluorescent proteins [34]. They nicely showed that PAPs are plastic structures that can engage and disengage from a dendritic spine in hippocampal slices. PAP motility have been confirmed by other researchers in hippocampus ([35]; see also Figure 2) as well as in BG contacting PC in acute slices of cerebellum [54, 55].

Time-lapse confocal studies have definitively demonstrated that PAPs are highly dynamic as they can modify their structure in a time course of minutes. PAP movements were observed in primary and organotypic cultures as well as in the acute slice model. Whether PAP motility occurs only during development and synaptogenesis or is persistent in adulthood and how this remodeling occurs *in vivo* remain, however, to be determined.

## 5. Driving Force and Mechanisms of PAP Structural Plasticity

Already early EM observations of astrocytic structural plasticity in rat hypothalamus suggested a dependence on environmental cues and stimulation, such as parturition, lactation, osmotic stimulation, and stress [44–48]. An EM study in the visual cortex of rats reared in a complex environment revealed a specific and significant increase in the ensheathment of synapses by astrocytic processes that accompanied structural and functional synaptic changes [56]. Chronic whisker stimulation in adult mice induced PAP structural plasticity in the corresponding barrel of the somatosensory cortex. Serial EM analyses have revealed that stimulation leads to a significant increase of the perimeter of excitatory synapse covered by PAPs, particularly at the axon-spine interface [41]. In dentate gyrus of hippocampus, the spatial relationship between astrocytic processes and synapses was analyzed at different time-points after induction of long-term potentiation (LTP) in adult rats *in vivo* [57]. This study reported a significant increase in the number and surface density of astrocytic processes 8 h after induction of LTP. At this time-point, synaptic complexes became increasingly more enveloped by PAPs. In organotypic hippocampal slice cultures, the morphological remodeling of CA1 excitatory spine synapses underlying induction of synaptic potentiation by theta burst stimulation of Schaffer collaterals was accompanied by an increase in the glial coverage of both pre- and postsynaptic elements, particularly of the large spine synapses with complex PSDs. Importantly, the increase in coverage was NMDA receptor-dependent [28]. In the same hippocampal pathway, a growth of astrocytic processes related to kindling and ischemic preconditioning has also been reported [58]. Additionally, EM studies in both hippocampus and cortex revealed that PAPs are preferentially present near synapses with big and often complex-shaped PSDs that are considered as morphological correlates of strengthened synapses [27–29]. These data, together with an observation that the proportion of cortical astrocyte coverage is correlated with the size of the PSD [41] also strongly suggest that PAP structural organization can be regulated by synaptic activity. The EM data available so far show that structural and functional pre- and postsynaptic changes due to experience-related paradigms and LTP (read [59]) are accompanied by structural changes in adjacent PAPs.

PAP movements observed with time-lapse confocal microscopy in hippocampal slices appeared to be coordinated with the extent of spine movements suggesting that the two phenomena are linked [34]. Because spine dynamics is dependent upon neuronal activity, the same assumption could then be made for PAPs. Interestingly, by assessing PAP movements around spines using an index of motility, Haber and coworkers revealed a high heterogeneity of this parameter [34], reminiscent of the synaptic coverage heterogeneity described above. This could mean that synaptic coverage is intimately linked to PAP motility. The fast motility of PAPs could then represent the movements accomplished by PAPs to increase or decrease synaptic coverage.

Increase of neuronal activity in hippocampal slices by application of the GABAR inhibitor bicuculline failed to elevate PAP motility [34], and PAP movements still occurred in the presence of TTX [35]. Nevertheless, glutamate application induced PAP remodeling, and inhibition of glutamate transporter under TTX prevented coordinated PAP-spine movements, suggesting, as shown in the cultures, that glutamate could be involved in PAP structural plasticity *in situ* [35].

Other indications also suggest an activity-dependence of PAP structural plasticity. A well-established feature of astrocytes is their ability to respond with an intracellular calcium elevation (Ca^2+^_i_) to neurotransmitters such as glutamate [50], GABA [60], ATP [61], endocanabinoids [62], and others. This increase can be evoked by neuronal activity both *in situ* [63] and *in vivo* [64, 65]. Glutamate stimulates filopodia outgrowth on cultured astrocytes and these effects are mimicked by both kainate and quisqualate agonists [50], as well as mGluR agonist [4]. mGluR antagonists abolish this structural remodeling, while an NMDAR agonist [50] does not influence it. Astrocytes were shown to express Ca^2+^-permeable AMPAR [66, 67] and mGluR [68], the latter specifically in PAPs [4]. This suggests the involvement of non-NMDA ionotropic glutamate receptors (possibly AMPAR) and mGluR in the mechanisms driving astrocytic filopodia outgrowth. Consistent with this, BG retracted from PC spines when Ca^2+^-impermeant AMPAR were overexpressed in BG [54]. However, it should be mentioned that the subunit composition of AMPAR is regulated developmentally, with Ca^2+^-permeable AMPAR subunits (GluA2) decreasing during development [69]. Moreover, the question about the glial cell type that expresses AMPAR is still open, as NG2 cells and astrocytes share common GFAP marker [70]. mGluR 3 and 5 subtypes are localized in both filopodia from cultured astrocytes and in PAPs of hippocampal astrocytes *in situ* [4].

Although astrocytes are lacking voltage-gated channels, are not electrically excitable, and cannot generate action potentials, they can sense neuronal activity through calcium excitability, particularly thanks to the close apposition of PAPs to synapses [9, 10]. The most widely accepted mechanism for Ca^2+^_i_ signaling is the Gq protein coupled receptor-induced calcium release from intracellular stores, specifically from the endoplasmic reticulum [71]. Interestingly, calcium could be central to the mechanisms that control actin movements in astrocytes in the vicinity of the synapse [24, 72]. Calcium uncaging experiments in astrocytic cultures confirmed that Ca^2+^ elevation triggers filopodia outgrowth [24]. In acute slices from transgenic mice that have an attenuated IP_3_ pathway, kinetics of Ca^2+^ signals in hippocampal astrocytic processes following mGluR agonist application was reduced. EM analysis of the hippocampus from these mice revealed reduced PAP coverage of synapses as well as an elevated proportion of uncovered synapses [72]. This suggests that IP_3_-mediated Ca^2+^ signals may be part of the mechanism driving morphological changes in PAPs.

Actin-based movements and remodeling are sensitive to Ca^2+^ changes which are affecting a plethora of actin binding proteins. Fine astrocytic processes contain mostly actin cytoskeleton, and the actin-binding protein alpha-actinin aggregates at the tip of growing filopodia in cultured astrocytes [22]. Inhibitors of actin polymerization block PAP motility [34]. Filopodia growth requires the redistribution of cytoskeletal proteins but no *de novo* synthesis or degradation of respective proteins [22], suggesting that astrocytes are capable of rapid movements in response to their environment. In both cultures [22] and slices [52, 53], astrocytic processes can adapt their morphology through a mechanism involving the rac-1 member of the ras superfamily of GTPases. Small GTPases are also involved in the motility of Bergman glia in the cerebellum [55]. Interestingly, a dominant negative form of the actin binding protein profilin-1 abolished Ca^2+^-dependent filopodia outgrowth and movement [24]. Immunocytochemistry in rat brain sections demonstrated that the actin-binding protein ezrin and the mGluRs 3 and 5 are compartmentalized to PAPs but not to the main processes containing GFAP [4]. The experiments using ezrin siRNA or dominant-negative ezrin in primary astrocytes indicated that filopodia formation and motility require ezrin [4].

Astrocytic processes are subject to swelling (see review [13]). Even if swelling is often associated with pathological conditions, subtle volumetric changes can occur under physiological conditions, typically during osmotic pressure changes following synaptic transmission [73]. Thus, the swelling- and actin-based mechanisms for PAP structural plasticity could occur simultaneously [13].

Recent data indicate also the existence of extracellular mechanisms that could influence synapse-related PAP plasticity. Several astrocyte-neuron adhesion molecules were described, although there is still a lack of information about the presence of these molecules at the PAP-synapse interface. Among astrocyte-neuron adhesion molecules that could be regulated by activity and potentially localized at synapses the best candidate is the EphA4 receptor tyrosine kinase that is enriched in dendritic spines of hippocampal pyramidal neurons [74] (reviewed in [75]). The EphA4 ligand, ephrin-A3, appears to be localized on PAP membranes [76]. Binding of glial ephrin-A3 to neuronal EphA4 maintains normal spine morphology [76] while preventing the interaction results in unstable spines with disrupted shapes [53]. Interestingly, exogenous and endogenous ephrin-A induce astrocytic processes outgrowth and extension through their binding to astrocytic EphA ligands [52]. Concomitantly, calcium signals in astrocytes are perturbed by EphA, suggesting the implication of intracellular calcium in the mechanism regulating EphA4/ephrin-A3 adhesion and the subsequent regulation of individual dendritic spines.

Neuroligins are adhesion molecules known to be associated with synapses [77]. The expression of neuroligins 1 and 2 in the CNS is restricted to excitatory and inhibitory synapses, respectively. Neuroligin 3 is expressed by neurons but also by glial cells, in particular in the ensheathing glia in olfactory bulb as well as in retinal astrocytes [78]. Mutations of the genes coding for neuroligins and neurexins are associated with autism [79], a brain disorder characterized by anomalies of dendritic spine morphology. It was shown recently that astrocytes in autistic patients also exhibit significantly altered morphology, particularly of their processes. In addition, astrocytes of the neuroligin knockdown mice showed similar alterations [80]. This is suggesting implication of neuroligins in the maintenance of synaptic structures, including the astrocytic component. Very little is known about intracellular interaction partners of neuroligin 3. However, at glutamatergic synapses, neuroligin 1 binds to postsynaptic scaffolding protein PSD-95 that links to GKAP which in turn binds Shank, and this complex may further recruit other postsynaptic proteins to the excitatory synaptic junctions (read [81] for a review on neuroligins). Typically, mGluR receptors as well as ionotropic glutamate receptors are associated with Shank at postsynaptic sites, thereby providing a functional link between synaptic activity, intracellular calcium, and neuroligins (read [82] for a review on Shank).

Another potential candidate is the neuron-glia cell adhesion molecule (Ng-CAM). Ng-CAM has been implicated in binding between neurons and between neurons and glia [83]. SynCAM1, an adhesion molecule involved in synaptic differentiation and organization, is also expressed in astroglial cells where it mediates astrocyte-to-astrocyte and glial-neuronal adhesive communication [84]. NCAM is probably the most extensively studied cell adhesion molecule and is thought to intervene in most cell interactions via modulation of cell adhesivity and intracellular signaling. It is especially conspicuous in hypothalamo-neurohypophysial astrocytes (reviewed in [85]).

N-cadherin adhesion molecule is implicated in the establishment and stability of neuronal connections through activity-dependent mechanisms [86, 87]. The most important mechanism is the modification in extracellular calcium concentration, which affects the structure of the ectodomain and cadherin-mediated cell adhesion. Also, the cytoplasmic domain binds to various cytosolic and membrane proteins, including heterotrimeric G proteins. Moreover, the C-terminal domain mainly binds to catenin and anchors the protein to the actin cytoskeleton [88]. Interestingly, a member of the cadherin family, protocadherin-gammaC5 that is implicated in activity-regulated synaptic stability, is expressed in both astrocytes and neurons. In the brain, significant numbers of Pcdh-gammaC5 clusters are located at contact points between neuronal synaptic components and PAPs, suggesting that Pcdh-gammaC5 is involved in neuron-astrocyte interactions at the synaptic level [89].

Together, these data suggest that perisynaptic astrocytic processes possess the machinery to both sense neuronal activity and remodel their actin filaments in an activity-dependent manner. These mechanisms are regulated by intracellular calcium, through the IP3 pathway, and could directly contribute to activity-dependent PAP structural plasticity. The data on adhesion molecules expressed in PAPs suggest their participation in remodeling, repositioning, and stabilization of perisynaptic astrocytic arrangements, although further research is needed to elucidate their exact roles.

## 6. What Are the Consequences for a Synapse?

Astrocytic ensheathment of the synapses may constitute a physical barrier for transmitter spillover from the synaptic cleft leading to extra- and heterosynaptic signaling. The consequences of the heterogeneity of the synaptic coverage by PAPs observed so far in different brain regions have been discussed in several studies. This heterogeneity suggests that glutamate or other neurotransmitters escape nonuniformly from synapses in a given brain area and that at certain synapses and/or under certain conditions synaptic crosstalk will be favored through a better spillover [40]. Structural plasticity of PAPs might significantly contribute to these mechanisms. A good example is provided by the rapid and reversible structural remodeling of astrocytes in hypothalamus under conditions of stimulation, leading to a reduced coverage of the somata and dendrites of magnocellular secretory neurons [46, 48]. These anatomical modifications result in astrocytic-dependent synaptic metaplasticity [90]. For example, in the SON of lactating rats astrocytic coverage undergoes extensive reduction that leads to an increase in directly juxtaposed surfaces of OXT magnocellular neurons [91, 92] and the change of both tortuosity and volume fraction of ECS [93]. Consequently, the number of synaptic contacts is increased and diffusion of neuroactive substances is facilitated. At the same time, glutamate clearance is delayed resulting in an increased negative feedback of glutamate on its own release and facilitated heterosynaptic depression of GABA release through glutamate spillover [93, 94]. Also, astrocytes in SON provide D-serine, an endogenous ligand for NMDARs, so that astrocytic withdrawal in lactating animals leads to a decrease in synaptic responses mediated by NMDARs and in availability of these receptors for activation, thus influencing the direction and magnitude of long-term synaptic plasticity [95]. Similar structural modifications of neuronal-glial interactions under conditions of enhanced neurosecretion occur in parallel in the neurohypophysis, the major projection site of magnocellular neurons, leading to facilitation of hormone release to blood circulation [46].

In the SCN of the hypothalamus, EM analysis of glial and axonal coverage of the somata and dendrites of the neurons expressing vasoactive intestinal peptide (VIP) or arginine vasopressin (AVP), the two main sources of SCN efferents, revealed significant variations during the L/D cycle [96]. The glial coverage of VIP dendrites increases at night, in concomitance with a decrease in the coverage of the somata and dendrites of these neurons by axon terminals. Conversely, glial coverage of the AVP dendrites drops during nighttime with no change in axonal coverage but with a striking increase in the somatal and dendritic appositions thus helping nocturnal facilitation of the intercellular synchronization involving these neurons [49].

Synapse modeling studies focused on the glial perisynaptic environment suggested profound effects of glial coverage on activation profiles of perisynaptic receptors. Rusakov has estimated that if one-half of the synaptic perimeter is covered with PAPs, it leads to almost a twofold increase of the glutamate concentration inside the glial sheath and two- to fourfold decrease outside. If around 95% of the synapse is covered, the concentration difference between inner and outer sides can reach 10- to 100-fold. Synaptic coverage may have stronger effects on synaptic transmission and Ca^2+^ depletion in the cleft at smaller synapses, especially with slower kinetics of perisynaptic ion transients [97]. Obviously, modifications of synaptic coverage due to PAP structural plasticity may have significant impact on spillover effects. Quantitative topological analysis of the extracellular space (ECS) based on 3D EM reconstructions of the CA1 neuropil of the adult rat allowed to decompose ECS into sheets and tunnels that are built by the surface patches of the axonal/dendritic/glial membranes [98]. These sheets and tunnels are distributed nonuniformly, with axons surrounded by more tunnel-like ESC, astrocytic processes accompanied by more sheet-like volumes, while around dendritic spines, the ECS is more tunnel-like than elsewhere. Diffusion simulations indicated that release of bioactive molecules to ECS would produce higher concentration peaks and maximum rate of concentration change, as seen by cell surface receptors, in the narrower sheet regions than in the larger tunnel spaces. These spatial irregularities would imply that ECS sheets may be specialized into enhancing signaling through concentration changes, while volume transmission of large molecules would be ensured through tunnels. Given that glia swelling under different physiological and pathological conditions may modulate extracellular volume fraction and that astrocytic processes are preferentially accompanied by ECS sheets, astroglia are well positioned to regulate volume communication in neuropil and provide a selective and specialized control of perisynaptic ECS volume around a synapse [98].

As shown by light and EM immunochemistry, perisynaptic astrocytic processes (PAPs) surrounding excitatory spine synapses express a plethora of different proteins, among which glutamine synthetase [99], astrocytic glutamate transporters [100], and aquaporins that may regulate “adaptive” swelling of PAPs [101], as well as actin associated molecules [4, 23], metabotropic glutamate receptors [4], and cell adhesion molecules [46]. Thus, by enwrapping a synapse, astroglia not only constitute a physical barrier for transmitter diffusion and help to retain a high level of neurotransmitter around a synapse but also, through the expression of specific proteins in perisynaptic processes, can participate in both sensing and responding to synaptic activity, actively take up glutamate from the synaptic cleft [5], control the amount of glutamate spillover that activates extrasynaptic receptors or enable inter-synaptic crosstalk [6], buffer potassium following neuronal depolarization [102], and provide energy substrates to neurons [103]. Remarkably, astrocytes are also able to release so-called gliotransmitters in a calcium-dependent manner, among which are D-serine [104], glutamate [105], and ATP [106]. There is now extensive evidence that astrocytes can modulate synaptic transmission and plasticity through gliotransmission [62, 95, 107–115]. However, the exact mechanisms taking place during calcium-dependent neuron-glia interactions are still a matter of debate as studies using different approaches to selectively increase astrocytic Ca^2+^_i_ yielded conflicting results [12, 116, 117]. Gliotransmission is not the only consequence of calcium events in astrocytes. Calcium can regulate the expression level of glutamate transporters on astrocytes [118]. Intracellular calcium is also known to take part in the mechanism of neurometabolic coupling with neurons by either modulating the energetic response [119] or participating to the propagation of metabolic signals across the astrocytic network [120]. As discussed above, calcium signaling, often related to synaptic activity, could be central to the mechanisms that control PAP remodeling in the vicinity of the synapse [24, 72], which could in turn optimize the positioning of glial glutamate transporters to provide more efficient clearance of glutamate [5], modify the positioning of the release sites of gliotransmitters close to extrasynaptic neuronal receptors [2] or release of energetic substrates such as lactate in front of neuronal monocarboxylate transporters [121], and regulate ionic content of the extrasynaptic space [122].

Another aspect of PAP structural plasticity is its possible impact on the synaptic structure [40]. In concert with this concept, it was shown that in hippocampal slices the probability of a spine to disappear is higher when PAPs are not present around it [53]. Inhibition of astrocytic processes motility through interference with Rac1 results in an increase in dendritic filopodia [53], the protrusions known to be immature and nonpersistent [123]. Interfering with EphA4 or ephrin-A3 induces growth of astrocytic processes in cell culture [52]. The same treatments in slice cultures perturb the contact-dependent ephrin/Eph signaling by astrocytes, shorten the lifetime of dendritic protrusions contacted by astrocytic processes, and decrease the probability for the newly formed spines to be stabilized [53]. Consistent with this, BG coverage of PC spines seems to be required for synapse formation and regulation [54, 55]. Some hippocampal dendritic spines are able to extend spine head protrusions to neighboring functional presynaptic boutons [124]. Interestingly, the volume overlap between spines and PAPs decreased during the formation of spine head protrusion, suggesting an inverse correlation between spine coverage by PAPs and the creation of new synaptic connections [35]. These data point at the role of PAPs in regulation of new spine stability.

## 7. Discussion

Astroglia show intricate morphological interactions with neurons. Astrocytes are well positioned to influence synaptic transmission as they send fine processes that ensheath a synapse, forming a tripartite complex with pre- and postsynaptic neuronal components. About 60% of neuronal synaptic structures are partially enwrapped by PAPs, and the level of synaptic coverage increases with neuronal activity, LTP, and during experience-related paradigms. Moreover, PAPs can display rapid remodeling by extending and retracting from dendritic spines in a time scale of minutes. This rapid astrocytic motility appears to be influenced by neuronal activity and is coordinated with morphological changes in spines. In hippocampus, cortex, and cerebellum, glial processes exhibit two distinct structural plasticity phenomena: a highly dynamic movement referred to as motility and changes in their coverage of a synapse. Whether these two forms of plasticity are linked is still unclear, as well as the precise impact of such structural plasticity on synaptic function, structure, and plasticity.

One hypothesis that we would like to propose is that the activity-dependent structural plasticity of PAPs may allow astrocytes to control both the structural and functional properties of the synapse. Astrocytic contacts around synapses may provide structural support to a synapse while controlling the diffusion of molecules within the ECS and participating in extracellular homeostasis. Astrocytic processes in the vicinity of the synapse may control the amount of glutamate spillover that activates extrasynaptic receptors, enabling intersynaptic crosstalk and allowing for modified or synchronized neurotransmission. Similarly, it could help to locally provide energy substrates to neurons during energy demanding processes such as plasticity.

There are however many questions that still remain concerning the mechanisms of PAP structural plasticity, about how it is governed and controlled. Recent data provide indications that it could be controlled by neuronal activity, probably through glial metabotropic receptors and intracellular calcium. As Ca^2+^ signaling in astrocytic processes is synaptically evoked and can occur locally in the perisynaptic astrocytic environment without propagating to the cell body or other processes, PAP motility might be required to control the established “tripartite” connections in place and to coordinate their plastic adaptations [10]. Moreover, synaptic calcium signals in astrocytes associated with PAP structural plasticity could indicate that a given astrocyte can differentiate between the around 100 000 synapses it is enwrapping [17]. Thus, PAPs could be seen as structural as well as functional [125] entities able to integrate synaptic signals.

In the CNS, excitatory synapses exhibit multiple forms of plasticity that play a central role in information processing by neural networks. They involve changes in synaptic strength but also structural reorganization of synaptic connections that can occur during the whole lifetime [126]. These mechanisms of synaptic structural plasticity are now believed to be crucial for memory processes in the CNS. Consistent with this, abnormal synaptic morphologies are linked to various developmental psychiatric diseases including mental retardation, schizophrenia, and autism [127]. An important question therefore is to better understand the mechanisms regulating these structural aspects of plasticity. As PAP remodelling seems to be important for synapse function, formation, and stability, astrocytes might play an important role in these processes. Further studies, however, are needed to evaluate the functional and structural consequences of PAP structural plasticity in CNS function and disease.

## Abbreviations

3D: Three dimensional
ASI: Axon-spine interface
AVP: Arginine vasopressin
AMPA: 2-Amino-3-(3-hydroxy-5-methyl-isoxazol-4-yl)propanoic acid
AMPAR: AMPA receptor
BG: Bergman glia
Ca^2+^_i_: Intracellular calcium concentration
CF: Climbing fibers
ECS: Extracellular space
EM: Electron microscopy
EphA: Ephrin-A receptor
EphB: Ephrin-B receptor
GABA: Gamma-aminobutyric acid
GABAR: GABA receptor
GFAP: Glial fibrillary acidic protein
GFP: Green fluorescent protein
GKAP: Guanylate kinase-associated protein
L/D cycle: Light/dark cycle
LTP: Long-term potentiation
MF: Mossy fibers
mGluR: Metabotropic glutamate receptor
NMDA: n-Methyl-D-aspartic acid
NMDAR: NMDA receptor
NTS: Nucleus Tractus Solitarii
OXT: Oxytocin
PAP: Perisynaptic astrocytic process
PC: Purkinje cell
PF: Parallel fibers
PPC: Posterior piriform cortex
PVN: Paraventricular nucleus of hypothalamus
SAA: Surface-associated astrocytes
SCN: Suprachiasmatic nucleus of the hypothalamus
SON: Supraoptic nucleus of hypothalamus
VIP: Vasoactive intestinal peptide.
